# Reduced-cost two-level surrogate antenna modeling using domain confinement and response features

**DOI:** 10.1038/s41598-022-08710-2

**Published:** 2022-03-18

**Authors:** Anna Pietrenko-Dabrowska, Slawomir Koziel, Ubaid Ullah

**Affiliations:** 1 Faculty of Electronics, Telecommunications and Informatics, Gdansk University of Technology, 80-233 Gdansk, Poland; 2 Engineering Optimization and Modeling Center, Reykjavik University, 102 Reykjavik, Iceland; 3 Al Ain University, P.O. Box 112612, Abu Dhabi, UAE

**Keywords:** Electrical and electronic engineering, Computational science

## Abstract

Electromagnetic (EM) simulation tools have become indispensable in the design of contemporary antennas. Still, the major setback of EM-driven design is the associated computational overhead. This is because a single full-wave simulation may take from dozens of seconds up to several hours, thus, the cost of solving design tasks that involve multiple EM analyses may turn unmanageable. This is where faster system representations (surrogates) come into play. Replacing expensive EM-based evaluations by cheap yet accurate metamodels seems to be an attractive solution. Still, in antenna design, application of surrogate models is hindered by the curse of dimensionality. A practical workaround has been offered by the recently reported reference-design-free constrained modeling techniques that restrict the metamodel domain to the parameter space region encompassing high-quality designs. Therein, the domain is established using only a handful of EM-simulations. This paper proposes a novel modeling technique, which incorporates the response feature technology into the constrained modeling framework. Our methodology allows for rendering accurate surrogates using exceptionally small training data sets, at the expense of reducing the generality of the modeling procedure to antennas that exhibit consistent shape of input characteristics. The proposed technique can be employed in other fields that employ costly simulation models (e.g., mechanical or aerospace engineering).

## Introduction

Over the last few decades, various types of computational models have been extensively used in engineering design. They are more reliable than simpler representations due to the ability to adequately quantify various physical phenomena that impact the system operation in the most significant manner. Concurrent rapid development of computer hardware and simulation methods allowed for devising sophisticated commercial simulation software packages that are nowadays utilized in various fields, such as mechanical^1^, aerospace engineering^2^, or multi-physics domains^3^. As a consequence, it has become possible to evaluate truly complex large-scale systems, e.g., civil aircrafts^4^, ships over a random sea surface^5^ or airflow through wind turbines^6^. Despite being time-consuming to evaluate, simulation models permit to reduce the cost of prototyping. A representative example of an engineering field, where costly simulation models are largely employed, is antenna design.

Design of modern antenna systems faces numerous challenges, partially related to the increase of performance requirements, which may be attributed to emerging application areas such as internet of things^7,8^, 5G wireless communications^9,10^, medical imaging^11^, remote sensing^12^, as well as wearable^13^ or implantable devices^14^. Furthermore, the designers have to address the demands for additional functionalities, including multi-band^15^ or MIMO operation^16^, circular polarization^17^, polarization/pattern diversity^18^, and band-notch operation^19^. Physical space limitations and the resulting miniaturization trends constitute yet another challenge^20,21^. Fulfilling the aforementioned requirements makes antenna design an intricate task, and more often than not leads to an increased complexity of the antenna topologies.

Enlarged numbers of geometry parameters is a by-product, but a troublesome one. Reliable evaluation of complex structures can only be carried out using electromagnetic (EM) analysis because simpler representations (e.g., equivalent network models) are either unavailable or incapable of providing sufficient accuracy. The lack or insufficiency of theoretical tools makes EM-driven design imperative. However, even a single full-wave analysis of a geometrically involved antenna may be CPU demanding. Therefore, the computational cost of such EM-based procedures as numerical optimization^22^ or uncertainty quantification^23^, that require executing repetitive EM simulations, is often unacceptably high.

One of the possible ways of reducing the computational overhead related to the aforementioned design tasks is to replace the EM model with its cheaper representation (surrogate or metamodel)^24,25^. In surrogate-assisted optimization, the said representation is employed as a prediction tool that routes the search process toward the optimum solution at a negligible cost. The two major classes of metamodels exist, physics-based and data-driven (or approximation) ones. The former typically involves an underlying low-fidelity model, the construction of which relies on problem-specific knowledge. In antenna design, various physics-based techniques have been developed, including space mapping^26,27^, response correction algorithms^28^, adaptive response scaling^29^, cognition-driven design^30^, or shape-preserving response prediction^31^. The practical obstacle for this class of methods are limited options for low-fidelity modeling, which, in most cases, incorporate coarse-mesh EM simulations. This generally degrades the efficacy of the surrogate-assisted optimization frameworks to a large extent.

In the light of the mentioned issues, a more favorable choice seem to be approximation surrogates. The abundance of techniques exploiting data-driven surrogates, along with an easy access through various third-party toolboxes (DACE^32^, SUMO^33^, UQLab^34^) made them a common choice in modeling and design of antenna structures. As a matter of fact, the list of advantages of data-driven surrogates is considerably longer: low evaluation cost, flexibility, no need for physical insight into the system under design, and transferability between various application areas. Some of widely used techniques of this class include kriging^35,36^, radial basis functions (RBF)^37^, Gaussian process regression (GPR)^38^, support vector regression^39^, and neural networks in many variations^40,41^. Still, a construction of data-driven surrogates for modern antennas featuring intricate topologies and large numbers of geometry parameters is challenging. The two main obstacles include the curse of dimensionality (i.e., a rapid increase in cardinality of training data sets as a function of the number of antenna parameters and their ranges^42^), as well as strong nonlinearity of antenna responses as a function of both geometry parameters and frequency. As a consequence, data-driven modeling of antenna structures using conventional techniques^43,44^ is limited to relatively simple structures over low dimensional parameter spaces. Some of the mentioned issues may be addressed using methods such as high-dimensional model representation (HDMR)^45^ or orthogonal matching pursuit^46^, yet, these solutions are not applicable to general purpose modeling.

An entirely different approach to handling dimensionality issues has been offered by the recently proposed performance-driven modeling frameworks^47–49^. The key concept of the methods belonging to this class is to restrict the metamodel domain to the most promising region of the parameter space, which encompasses the designs of high-quality with respect to the assumed performance figures. The domain defined according to this paradigm has a significantly smaller volume than the traditional, box-constrained domain delimited by the lower and upper bounds on design variables. As a consequence, the computational overhead of setting up a surrogate therein is considerably smaller than within the conventional approach. Notwithstanding, in early versions of performance-driven modeling techniques (i.e., triangulation-based constrained modeling^47^ and the nested kriging^48^), surrogate domain definition involved a set of so-called reference designs: pre-optimized with respect to the selected combinations of the figures of interest and/or material parameters relevant to the particular design task. Clearly, the acquisition of the reference designs incurred substantial computational expenditures, which, in some cases, could be justified by multiple use of the framework, e.g., for redesigning the structure at hand for different operating conditions. This expenses have been largely reduced (by a factor of sixty percent) in the further advancement of the technique, i.e., reference-design-free constrained modeling^49^, where the constrained domain has been determined using a preselected set of random observables. Acquisition of these observables does not require solving any optimization tasks. Instead, a cost-efficient selection procedure is employed^49^, in which the decision on the acceptance of a specific observable is made using the knowledge extracted therefrom.

This paper proposes a novel surrogate modeling technique, which further improves the efficacy of the aforementioned reference-design-free constrained modeling method^49^ by incorporating the response features technology^50^. The key concept of our approach is to handle the modeling task at the level of the system response features (characteristic points), rather than the frequency characteristics in their entirety. This is motivated by the weakly nonlinear dependence between the feature point coordinates and design variables, in contrast to typically high nonlinearity of antenna characteristics (both as a function of geometry parameters and the frequency). The important benefit of incorporating the response features technology into the reference-design-free constrained modeling is a further and significant reduction of the training data acquisition cost. In our approach, the surrogate domain is defined in a cost-efficient manner following methodology proposed in Ref.^49^, whereas the surrogate is set up at the level of the antenna response features, which allows for smoothing out the functional landscape to be approximated. Joint exploitation of both methodologies allows for constructing metamodels of antenna input characteristics using data set of truly low cardinalities without compromising the modeling accuracy. Moreover, the surrogates set up using the proposed approach are valid within broad ranges of geometry and operating parameters. Our approach is benchmarked against conventional kriging modeling technique^26^, as well as the three state-of-the-art performance-driven modeling techniques, i.e., nested kriging^48^, reference-design-free constrained modeling^49^, and feature-based nested kriging technique^51^. As demonstrated using three antenna examples, the proposed technique is considerably more efficient than the benchmark procedures, allowing for achieving surrogate predictive power of less than one percent using as low as 230 data samples on average. This level of accuracy is beyond capacity of the conventional data-driven modeling technique even using 800 samples. In addition, the proposed technique outperforms other performance-driven techniques with the data acquisition cost reduced by the factor of eight (when rendering models of similar predictive power).

The main technical contributions of this work include:

- Incorporation of the response feature technology into reference-design-free constrained modeling framework,
- Constructing the surrogate at the level of the response features within a confined domain defined based on random observables,
- Demonstrating substantial computational savings as compared to the previously reported constrained modeling techniques,
- Demonstrating superiority over conventional data-driven modeling techniques in terms of the CPU cost and surrogate model accuracy.

According to the authors’ knowledge, no comparable modeling technique ensuring this level of accuracy at such a low computational cost has not been reported in the context of antenna modeling in the literature thus far.

### Two-level constrained modeling with response features

The purpose of this section is to introduce the proposed modeling framework. Our technique capitalizes on the concept of performance-driven modeling, specifically, reference-design-free constrained modeling^49^, as well as the response features technology^50^. “Constrained modeling: Concept and basic definitions” and “Reference-design-free constrained modeling” sections provide a recollection of the constrained modeling technique^49^. “Response features” section outlines the response feature methodology^50^, whose incorporation into the proposed modeling framework is discussed in “Constrained modeling at the level of response features”. The formulation of the complete two-level constrained modeling technique, accommodating both the aforementioned technologies, concludes the section.

### Constrained modeling: concept and basic definitions

We start by recollecting the performance-driven modeling concept^52^, employed here for a surrogate domain definition purposes. In short, the techniques belonging to this group^47–49^ aim at identifying the parameter space regions that encompass the designs of high-quality from the point of view of the relevant figures of interest. This allows for a significant reduction of the domain volume in comparison to the conventional one, i.e., delimited by the lower and upper bounds on the design variables. As a consequence, substantial savings in terms of training data acquisition cost may be achieved without degrading surrogate model predictive power. This is of paramount importance especially for higher-dimensional cases. At the same time, this is achieved without formally narrowing down the ranges of neither antenna geometry parameters nor operating conditions^47,48^.

Table 1 gathers the basic objects utilized in constrained modeling^48^: the design variable vector ***x*** (typically geometry parameters of the device under study), as well as the two spaces of interest: the parameter space *X*, and the objective space *F*. The entries of the design objective vector *F* may include, e.g., antenna operating frequency/frequencies or bandwidth/bandwidths, but also substrate permittivity the structure is implemented on. The region of validity of the surrogate is supposed to cover the objective space *F*, delimited by the user-specified ranges of performance figures.

**Table 1:** Constrained modeling: basic object definitions.

Description	Notation
Design variables vector	***x*** = [*x*_1_ … *x*_*n*_]^*T*^
Conventionally defined parameter space	*X* = [***l***, ***u***]
Lower bounds on the design variables	***l*** = [*l*_1_ …, *l*_*n*_]^*T*^
Upper bounds on the design variables	***u*** = [*u*_1_ …, *u*_*n*_]^*T*^
Performance figures	*f*_*k*_, *k* = 1, …, *N*
Objective space	*F*: *f*_*k.*min_ ≤ *f*_*k*_^(*j*)^ ≤ *f*_*k.*max_, *k* = 1, …, *N*
Objective vector	***f*** = [*f*_1_ … *f*_*N*_]^*T*^

In constrained modeling, the surrogate model domain is to encompass the designs optimal with respect to the assumed figures of merit. The optimal design is understood here as minimizing the scalar objective function *U*(***x***,***f***) that quantifies the design quality^52^

1 $${\varvec{x}}^{*} = U_{F} ({\varvec{f}}) = \arg \mathop {\min }\limits_{{\varvec{x}}} U({\varvec{x}},{\varvec{f}})$$

The set of designs optimal with respect to all the objective vectors ***f*** ∈ *F*, is denoted as *U*_*F*_(*F*) = {*U*_*F*_(***f***) : ***f**** ∈ F*}. The surrogate is to be set within a domain that constitutes the region of the parameter space adjacent to the manifold *U*_*F*_(*F*). In nested kriging^48^, this region has been identified with the use of the set of pre-optimized reference designs ***x***^(*j*)^ = [*x*_1_^(*j*)^ … *x*_*n*_^(*j*)^]^*T*^, *j* = 1, …, *p*, corresponding to the objective vectors ***f***^(*j*)^ = [*f*_1_^(*j*)^ … *f*_*N*_^(*j*)^]. The pairs {***f***^(*j*)^, ***x***^(*j*)^}, *j* = 1, …, *p*, constitute a training data set to set up a first-level interpolation surrogate ***s***_*I*_(***f***) : *F* → *X*, which served to render an initial approximation of the manifold *U*_*F*_(*F*).

Needless to say, acquisition of the reference designs is expensive in terms of a required number of full-wave EM-simulations. As a matter of fact, its overall cost has been typically as high as a several hundreds of EM analyses^28^. Furthermore, obtaining these designs required re-designing the antenna at hand over broad ranges of operating conditions. Therefore, the reference design acquisition has been laborious and difficult to automate. Recently, some attempts to make it less dependent on designer’s supervision have been reported^53^).

### Reference-design-free constrained modeling

In the improved-efficiency constrained modeling technique^49^, the reference designs acquisition is abandoned altogether. Instead, a set of random observables is distributed in the parameter space *X*. These observables undergo a pre-selection process, in which their acceptance or rejection is based on the information about the design objectives extracted therefrom. The approved observables serve to construct an inverse regression model (a counterpart of the first-level interpolation model of the nested kriging^48^) for surrogate domain definition.

Let {***x***_*r*_^(*j*)^, ***f***_*r*_^(*j*)^}, *j* = 1, 2, …, be a sequence of pairs containing random vectors ***x***_*r*_^(*j*)^ uniformly distributed in the design space *X*, as well as the corresponding performance figure vectors ***f***_*r*_^(*j*)^ (extracted from the antenna model responses at ***x***_*r*_^(*j*)^). The acceptance/rejection process is carried out as follows: the *j*th observable is accepted if ***f***_*r*_^(*j*)^ ∈ *F*; otherwise (i.e., if either of the components of ***f***_*r*_^(*j*)^ is not within the assumed ranges on the performance figures or it is unidentifiable) the observable is rejected. The sample acquisition continues until the required number of observables *N*_*r*_ has been acquired (typically, *N*_*r*_ should be around ten times higher than the parameter space dimensionality). The said data pairs serve as a training set for setting up the inverse surrogate *s*_*r*_^49^

2 $$s_{r} ({\varvec{f}}) = {\varvec{s}}_{r} \left( {[f_{1} \; \ldots \;f_{N} ]^{T} } \right) = \left[ \begin{gathered} s_{r.1} ({\varvec{f}}) \\ \cdots \\ s_{r.n} ({\varvec{f}}) \\ \end{gathered} \right] = \left[ \begin{gathered} a_{1.0} + a_{1.1} \exp \left( {\sum\limits_{k = 1}^{N} {a_{1.k + 1} f_{k} } } \right) \\ \cdots \\ a_{n.0} + a_{n.1} \exp \left( {\sum\limits_{k = 1}^{N} {a_{n.k + 1} f_{k} } } \right) \\ \end{gathered} \right]$$

**Figure 1:**
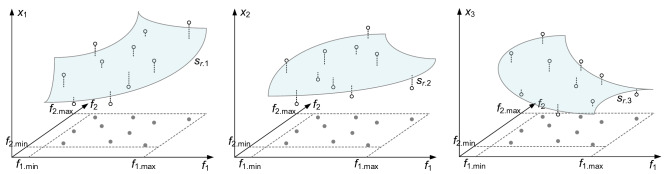
Visualization of the regression surrogate *s*_*r*_ constructed using the accepted observables ***x***_*r*_^(*j*)^ and their respective objective vectors ***f***_*r*_^(*j*)^: each component of *s*_*r*.*j*_, corresponding to a consecutive antenna parameter *x*_1_ (left), *x*_2_ (middle), and *x*_3_ (right), is shown as the grey-shaded manifold; black circles mark the observables, whereas black squares are their counterparts in the objective space.

Observe that (2) describes an inverse regression surrogate *s*_*r*_(***f***) that maps the antenna objective space into its design space. In other words, *s*_*r*_(***f***) is defined over the objective space F and assumes values in the parameter space *X*, or *s*_*r*_ : *F* → *X*. The above inverse model yields an approximation of the optimum design manifold *U*_*F*_. Identification of the surrogate *s*_*r*_ requires solving the following nonlinear regression problems

3 $$\left[ {a_{j.0} \;a_{j.1} \; \ldots \;a_{j.K + 1} } \right] = \arg \mathop {\min }\limits_{{[b_{0} \;b_{1} \; \ldots \;b_{K + 1} ]}} \sum\limits_{k = 1}^{{N_{r} }} {w_{k} \left[ {s_{r.j} \left( {{\varvec{f}}_{r}^{(k)} } \right) - x_{r.j}^{(k)} } \right]^{2} } ,\quad j = {1}, \ldots ,n$$

where *x*_*r.j*_^(*k*)^ denotes the *j*th entry of the observable vector ***x***_*r*_^(*k*)^, whereas the weighting factors *w*_*k*_ = [*w*_max_ – max{*p*_1_(***x***^(*j*)^), …, *p*_*N*_(***x***^(*j*)^)}]^2^, *k* = *1, …, N*_*r*_, differentiate “good” observables from the “poor” ones. We have the maximum factor *w*_max_ = max{*k* = 1, …, *N*_*r*_, *j* = 1, …, *N* : *p*_*j*_^(*k*)^}, with *p*_*j*_^(*k*)^ assuming nonnegative values (a better design is assigned a lower value of *p*_*j*_^(*k*)^). The factors *p*_*j*_^(*k*)^ are assembled into vectors ***p***_*r*_^(*j*)^ = [*p*_*r.*1_^(*j*)^ … *p*_*r.N*_^(*j*)^]^*T*^. The above mechanism of the weighted regression allows for ensuring that high-quality observables have more impact on the regression model, but also to take into account information contained in lower-quality ones. The vectors ***p***_*r*_^(*j*)^ are extracted from EM-simulated antenna response, similarly as the vectors ***f***_*r*_^(*j*)^. As an example, let us consider a dual-band antenna with the operating frequencies being the performance figures of interest. In this case, the vector ***f***_*r*_^(*j*)^ comprises the actual operating frequencies, whereas the vector ***p***_*r*_^(*j*)^ may contain the corresponding reflection levels. The concept of the inverse regression surrogate is visualized in Fig. 1.

In reference-design-free constrained modeling^49^, the surrogate domain definition procedure resembles that of the nested kriging technique^48^: the image of the inverse first-level surrogate *s*_*r*_(*F*) is extended in order to encompass the majority of the optimum design manifold *U*_*F*_(*F*). This is because *s*_*r*_(*F*) provides merely the initial inexact approximation of the location of *U*_*F*_(*F*). The coefficients of the said extension (towards the vectors normal to *s*_*r*_(*F*)) are given by

4 $${{\varvec{\upalpha}}}({\varvec{f}}) = [\alpha_{1} ({\varvec{f}})\; \ldots \;\alpha_{n - N} ({\varvec{f}})]^{T} = \left[ {|{\varvec{\tau v}}_{n}^{(1)} ({\varvec{f}})|\; \ldots \;|{\varvec{\tau v}}_{n}^{(n - N)} ({\varvec{f}})|} \right]^{T}$$

In (4), the orthonormal basis of vectors that are orthogonal to *s*_*r*_(*F*) at a given objective vector ***f*** is denoted as {***v***_*n*_^(*k*)^(***f***)}*, k* = 1, …, *n – N*. Moreover, a vector **τ** = [τ_1_ … τ_*n*_]^*T*^ comprises positive real numbers determining the amount of the extension. Having these, the surrogate model domain *X*_*S*_ is defined as

5 $$X_{S} = \left\{ \begin{gathered} {\varvec{x}} = s_{r} ({\varvec{f}}) + \sum\limits_{k = 1}^{n - N} {\varphi_{k} \alpha_{k} ({\varvec{f}}){\varvec{v}}_{n}^{(k)} ({\varvec{f}})} :{\varvec{f}} \in F,\; \hfill \\ \;\;\;\;\; - 1 \le \varphi_{k} \le 1,\;k = 1,...,n - N \hfill \\ \end{gathered} \right\}$$

In other words, the domain *X*_*S*_ encompasses all the vectors defined by (5) for all ***f*** ∈ *F*, and all the coefficients *φ*_*k*_ ∈ [–1, 1], *k* = 1, …, *n* – *N*. Thus, *X*_*S*_ is confined between the surfaces $S_{ + } = \left\{ {{\varvec{x}} \in X:{\varvec{x}} = s_{r} \left( {\varvec{f}} \right) + \sum\nolimits_{k = 1}^{n - N} {\alpha_{k} ({\varvec{f}}){\varvec{v}}_{n}^{(k)} ({\varvec{f}})} } \right\}$ and $S_{ - } = \left\{ {{\varvec{x}} \in X:{\varvec{x}} = s_{r} \left( {\varvec{f}} \right) - \sum\nolimits_{k = 1}^{n - N} {\alpha_{k} ({\varvec{f}}){\varvec{v}}_{n}^{(k)} ({\varvec{f}})} } \right\}$.

In reference-design free modeling technique^49^, the values of the extension factors are set individually for each design variable based on the knowledge extracted from the available observable set. For each observable pair {***x***_*r*_^(*j*)^,***f***_*r*_^(*j*)^}, a vector *P*_*k*_(***x***_*r*_^(*j*)^) minimizing the distance between the observable and [*s*_*r.k*_(***f***) ***f***^*T*^]^*T*^, ***f*** ∈ *F* is defined

6 $$P_{k} \left( {{\varvec{x}}_{r}^{(j)} } \right) = \arg \mathop {\min }\limits_{{{\varvec{f}} \in F}} \left\| {\left[ {x_{r.k}^{(j)} \;\left( {{\varvec{f}}_{r}^{(j)} } \right)^{T} } \right]^{T} - [s_{r} ({\varvec{f}})\;{\varvec{f}}^{T} ]^{T} } \right\|$$

The minimum distance between [*x*_*r.k*_^(*j*)^ (***f***_*r*_^(*j*)^)^*T*^]^*T*^ and the image of the *k*th component of the inverse regression model is *d*_*r*.*k*_(***x***_*r*_^(*j*)^) =||[***x***_*r.k*_^(*j*)^(***f***_*r*_^(*j*)^)^*T*^]^*T*^ – [*s*_*r*_(*P*(***x***_*r*_^(*j*)^)) *P*(***x***_*r*_^(*j*)^)^*T*^]^*T*^||. Thus, the extension factors *T*_*k*_ are given by^49^

7 $$T_{k} = \frac{1}{{2N_{r} }}\sum\limits_{j = 1}^{{N_{r} }} {d_{r.k} ({\varvec{x}}_{r}^{(j)} )} ,\quad k = {1}, \ldots ,n{-}N$$

All the factors are gathered in the extension vector ***T*** = [*T*_1 …_ *T*_*n–N*_]^*T*^.

The final, forward surrogate model ***s***(***x***) is set up in the confined domain *X*_*S*_ (defined using (4), (5) and (7)) as a kriging interpolation metamodel^32^ using the pairs {***x***_*B*_^(*k*)^,***R***(***x***_*B*_^(*k*)^)}_*k* = 1, …, *NB*_, where ***x***_*B*_^(*k*)^  ∈  *X*_*S*_ denote the training data samples, whereas ***R*** is the EM-simulated antenna response. The training data set is also complemented by the observable set {***x***_*r*_^(*l*)^,***R***(***x***_*r*_^(*l*)^)}_*l* = 1, …, *Nr*_.

### Response features

Modeling of highly-nonlinear antenna characteristics often proves to be a challenging task. In some cases, it is possible to reduce its complexity by employing the response feature technology^50^, where the modeling problem is tackled at the level of suitably defined characteristic points of the antenna at hand. The response feature technology^50^ capitalizes on a significantly less nonlinear relationship between the feature coordinates and designable parameters^54^ than normally observed for entire antenna responses. In the context of modeling, (but also parameter tuning^54^ or yield optimization^55^) this allows for a notable reduction of the computational overhead, which is of paramount importance, especially for devices described by larger (over ten) numbers of geometry parameters.

**Figure 2:**
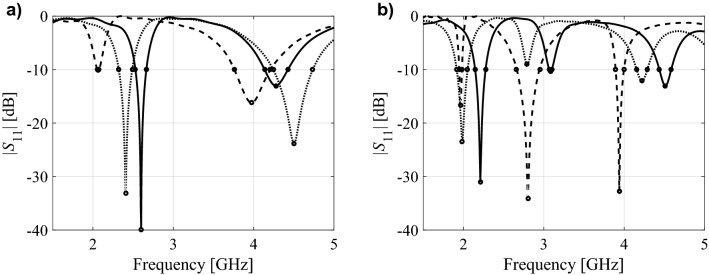
Exemplary antenna responses and their corresponding feature points marked with circles: (**a**) dual-band antenna, (**b**) triple-band antenna. Here, the response features include antenna resonances, as well as − 10 dB reflection levels; observe that not all the features can be distinguished for each antenna response shown in the pictures.

Clearly, the employment of response feature technology is only realizable when the system outputs are characterized by readily discernible characteristic points. From the practical point of view, the actual selection of response features has to account for the design goals. As an example, let us consider characteristics of a dual- and a triple-band antenna with characteristic points corresponding to antenna resonant frequencies and − 10 dB reflection levels, as shown in Fig. 2. These points permit handling design tasks aimed at resonance allocation at the target operating frequencies or bandwidth enhancement.

Naturally, the designer has to bear in mind that, in some cases, not all the characteristic points may be distinguished for a specific design, and to handle this issue appropriately during the optimization or modeling process. Technically, the response features are extracted from the EM-simulated antenna responses. Observe, that the feature-based approach permits to directly access information about the performance figures relevant to the assumed design objectives. This is in contrast to the conventional approach, where the entire antenna characteristics are handled, and this knowledge needs to be extracted afterwards. For a more thorough account of the response feature technology see, e.g., Ref.^52^.

### Constrained modeling at the level of response features

In the proposed modeling technique, the construction of the first-level regression surrogate *s*_*r*_, as well as domain definition procedure follow exactly that Ref^49^, which is recapitulated in “Reference-design-free constrained modeling”. Yet, unlike^49^, the second-level metamodel is set up using the training data pairs {***x***_*B*_^(*k*)^, ***F***_***R***_(***x***_*B*_^(*k*)^)}_*k* = 1, …, *NB*_, (along with the observables {***x***_*r*_^(*l*)^,***R***(***x***_*r*_^(*l*)^)}_*l* = 1, …, *Nr*_.). Here, ***F***_***R***_(***x***) = [*f*_1_(***x***) *f*_2_(***x***) … *f*_*p*_(***x***) *λ*_1_(***x***) *λ*_2_(***x***) … *λ*_*p*_(***x***)]^*T*^ denotes the response feature vector corresponding to a given design ***x***. The entries of the vector ***F***_***R***_ are the frequency *f*_*j*_ and level coordinates *λ*_*j*_, *j* = 1, …, *p*, of *p* antenna resonances. In other words, the response of the second-level surrogate yields predictions only about the feature point coordinates rather than the entire antenna characteristic at a given design ***x*** ∈ *X*_*S*_. Naturally, focusing only on the response features does lead to some unavoidable loss of information. Still, this loss is irrelevant from the point of view of the design goals: as mentioned in “Response features”, the features are defined so as to allow for quantifying the design objectives unequivocally. In general, some of the feature points may not be distinguishable (e.g., − 10 dB reflection points do not exist if the antenna resonance level is above that limit). However, this is not an issue for the considered approach, because the very definition of the domain *X*_*S*_ ensures that the antenna designs contained therein are of high quality, which ensures the existence of all feature points.

**Figure 3:**
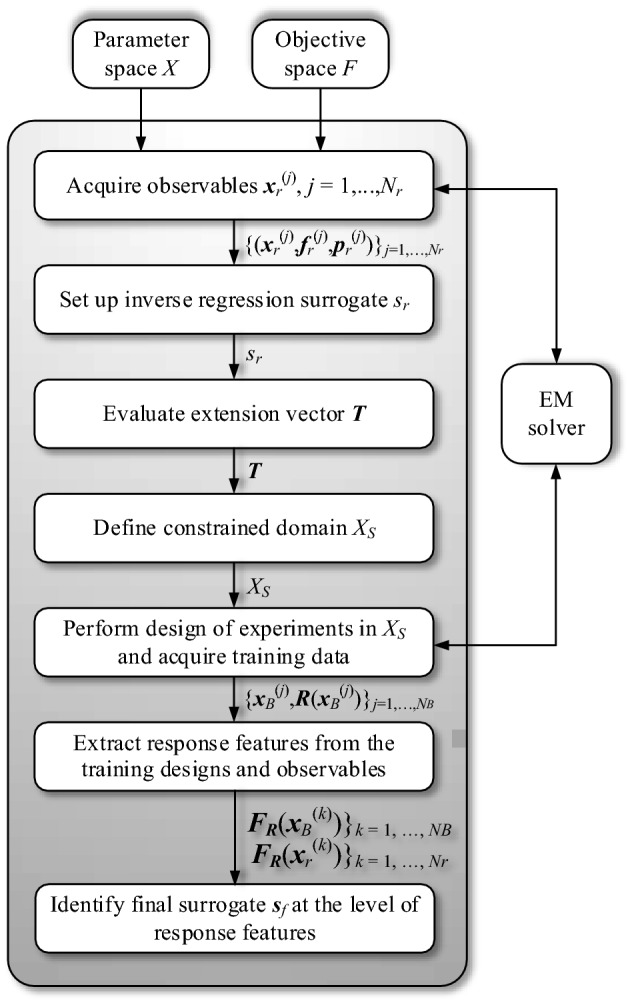
Flow diagram of the feature-based reference-design-free constrained modeling technique.

Figure 3 shows the conceptual illustration of the proposed modeling procedure. The user needs to define the parameter space and the objective space (by providing the respective lower and upper bounds), and also decide upon the number of the observables *N*_*r*_ (typically several dozen or so) that are to be used for domain definition purposes. Moreover, the number of the training data samples *N*_*B*_ for the construction of the final surrogate has to be selected. The modeling procedure consists of the following steps:

1. Acquisition of random observables ***x***_*r*_^(*j*)^  ∈ *X* (the process is terminated if the assumed number of *N*_*r*_ samples characterized by the objective vectors ***f***_*r*_^(*j*)^ belonging to the objective space *F* has been gathered), followed by the evaluation of auxiliary performance vectors ***p***_*r*_^(*j*)^ for the accepted samples;
2. Construction of the inverse regression surrogate with the training data pairs {***x***_*r*_^(*j*)^,***f***_*r*_^(*j*)^}_*j* = 1,…,*Nr*_, and the weighting factors assessed based on the vectors ***p***_*r*_^(*j*)^;
3. Evaluation of the extension vector ***T*** (cf. (6)–(7)), along with the extension coefficients **α** using (4);
4. Definition of the constrained domain *X*_*S*_ using (5);
5. Design of experiments: rendition of *N*_*B*_ training samples {***x***_*B*_^(*k*)^,***R***(***x***_*B*_^(*k*)^)}_*k* = 1, …, *NB*_;
6. Extraction of the features ***F***_***R***_(***x***_*B*_^(*k*)^)}_*k* = 1, …, *NB*_, from the training designs ***x***_*B*_^(*k*)^;
7. Extraction of the features ***F***_***R***_(***x***_*r*_^(*k*)^)}_*k* = 1, …, *Nr*_, from the observables ***x***_*r*_^(*k*)^;
8. Construction of the final surrogate model ***s***_*f*_ with the training data set being the extracted features from both the training samples and the observable set.

Let us recall the examples of previous Sections to underline how straightforward the incorporation of the response features technology into the feature-based reference-free constrained modeling framework is. The entries of the objective vectors ***f***_*r*_^(*j*)^ of “Reference-design-free constrained modeling” are the antenna operating frequencies, whereas the auxiliary performance vectors ***p***_*r*_^(*j*)^, utilized to quantify the quality of the observables, are the reflection levels at these frequencies. Observe that the first *p* entries of the feature vectors ***F***_*r*_^(*j*)^ of “Response features” (i.e., the frequency coordinates) coincide with the entries of ***f***_*r*_^(*j*)^. At the same time, the remaining *p* entries of ***F***_*r*_^(*j*)^ (i.e., the level coordinates) are simply the components of the performance vectors ***p***_*r*_^(*j*)^. The operating flow of the presented modeling procedure has been shown in Fig. 3.

## Results

This section provides numerical verification of the proposed modeling technique. The results have been obtained for three antenna structures and benchmarked against the conventional kriging interpolation, as well as the state-of-the-art constrained modeling frameworks: nested kriging, feature-based nested kriging, and reference-design-free modeling.

### Antenna structures used as verification cases

**Figure 4:**
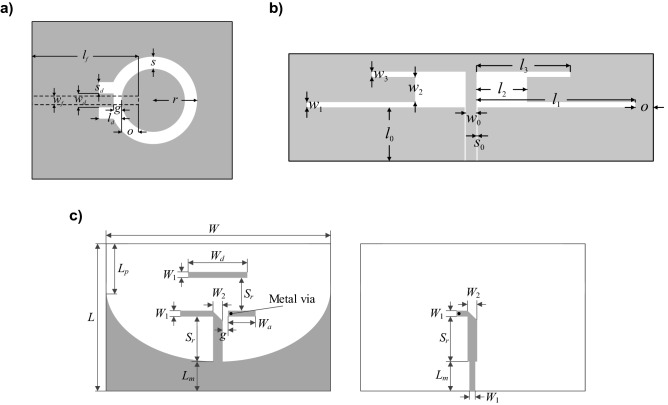
Geometries of the benchmark antenna structures: (**a**) ring slot antenna^56^ (Antenna I) with microstrip feed marked using a dashed line, (**b**) dual-band dipole antenna^57^ (Antenna II), and (**c**) quasi-Yagi antenna^58^ (Antenna III) top layer (left), and bottom layer (right).

The proposed modeling framework has been demonstrated using the following antenna structures: a ring-slot antenna^56^ (Antenna I), a dual-band dipole antenna^57^ (Antenna II), and a quasi-Yagi antenna^58^ (Antenna III) presented in Fig. 4a, b and c, respectively. The details concerning the design variables and objectives, as well as simulation models for all the benchmark structures have been gathered in Table 2. For Antenna I and III, the substrate relative permittivity *ε*_*r*_ is one of the performance figures, therefore, its ranges are provided under the design objective ranges of Table 2. The computational models are evaluated in CST Microwave Studio and simulated using its time-domain solver.

**Table 2:** Verification antenna structures.

Antenna	Antenna I	Antenna II	Antenna III
Substrate	*h* = 0.76 mm	*h* = 0.76 mm, *ε*_*r*_ = 3.5	*h* = 1.5 mm
Designable parameters	***x*** = [*l*_*f*_* l*_*d*_* w*_*d*_* r s s*_*d*_* o g*]^*T*^	***x*** = [*l*_1_ *l*_2_ *l*_3_ *w*_1_ *w*_2_ *w*_3_]^*T*^	***x*** = [*W L L*_*m*_* L*_*p*_* S*_*d*_* S*_*r*_* W*_2_ *W*_*a*_* W*_*d*_* g*]^*T*^
Other parameters (mm)	**–**	*l*_0_ = 30, *w*_0_ = 3, *s*_0_ = 0.15, *o* = 5	–
Lower bounds (mm)	***l*** = [22.0 3.5 0.3 6.5 3.0 0.5 3.5 0.2]^*T*^	***l*** = [29 5.0 17 0.2 1.5 0.5]^*T*^	***l*** = [100 55 10 14.5 6.0 10.0 2.0 7.5 16.3 0.5]^*T*^
Upper bounds (mm)	***u*** = [27.0 8.0 2.3 16.0 7.0 5.5 6.0 2.3]^*T*^	***u*** = [42 12 25 0.6 5.2 3.5]^*T*^	***u*** = [137 81 29 28 21 18 5.0 20 40 1.0]^*T*^
Fine model	~ 300,000 cells	~ 100,000 cells	~ 600,000 cells
LPW	20	20	20
Simulation accuracy	–40 dB	–35 dB	–30 dB
Simulation time	90 s	60 s	240 s
Design objectives	minimize the antenna reflection at *f*_0_	minimize the antenna reflection at *f*_1_ and *f*_2_	minimize the antenna reflection and enhance gain within 8-percent fractional bandwidth around *f*_0_
	**Design objective ranges**		
*F*_1_	Substrate permittivity	Operating frequency (lower band)	Substrate permittivity
	2.0 ≤ *ε*_*r*_ ≤ 5.0	2.0 GHz ≤ *f*_1_ ≤ 3.0 GHz	2.5 ≤ *ε*_*r*_ ≤ 4.5
*F*_2_	Operating frequency	Operating frequency (upper band)	Operating frequency
	2.5 GHz ≤ *f*_0_ ≤ 6.5 GHz	4.0 GHz ≤ *f*_2_ ≤ 5.5 GHz	2.5 GHz ≤ *f*_0_ ≤ 5.0 GHz

### Modeling results

The surrogate models have been constructed within the respective regions of validity given in Table 2, using the following sizes of the training data sets: 20, 50, 100, 200, 400, and 800 samples. The benchmark techniques include: (i) conventional kriging in an unconstrained domain^32^ (Algorithm 1), (ii) basic nested kriging technique^48^ (Algorithm 2), (iii) reference-design-free constrained modeling^49^ (Algorithm 3), (iv) feature-based nested kriging technique^51^ (Algorithm 4). The proposed feature-based reference-design-free constrained modeling framework is referred to as Algorithm 5. The main features of the frameworks considered in this paper are summarized in Table 3, where the surrogate model definition setup and costs are compared, along with the modeling task formulation: conventional (operating on the entire responses) or feature-based. Tables 4, 5 and 6 provide the modeling results: the computational costs of model setup and its accuracy. Observe that in the case of the feature-based performance-driven techniques (i.e., Algorithm 4, and the proposed Algorithm 5), the modeling accuracies of the frequency and level coordinates of the response features are provided.

**Table 3:** Constrained modeling: basic object definitions.

Modeling framework		Surrogate domain	Surrogate domain definition	Surrogate domain definition cost	Modeling task formulation
1	Conventional kriging^32^	Defined by the lower and upper bound on geometry parameters	N/A	N/A	Conventional
2	Nested kriging^48^	Constrained	Set up using a set of reference designs	Approx. 100*n*^$^	Conventional
3	Reference-design-free constrained modeling^49^	Constrained	Set up using random observables	Approx. 10*n*^$^	Conventional
4	Feature-based nested kriging^51^	Constrained	Set up using a set of reference designs	Approx. 100*n*^$^	Feature-based
5	Feature-based reference-design-free constrained modelling (This work)	Constrained	Set up using random observables	Approx. 10*n*^$^	Feature-based

^$^*n* denotes the number of the antenna geometry paramers.

**Table 4:** Ring-slot antenna of Fig. 4a: modeling results and benchmarking.

Number of training samples	Modeling technique											
	Kriging^32^		Nested kriging^48^		Reference-design-free modeling^49^		Feature-based nested kriging^51^			This work		
	Modeling error (%)	Model setup cost	Modeling error (%)	Model setup cost^$^	Modeling error (%)	Model setup cost^$^	Modeling error *f* (%)	Modeling error *l* (%)	Model setup cost^$^	Modeling error *f* (%)	Modeling error *l* (%)	Model setup cost
20	93.8	20	59.5	884	45.1	126	3.91	32.8	884	0.71	12.8	126
50	56.9	50	19.4	914	13.4	156	3.29	27.8	914	0.43	8.5	156
100	50.8	100	12.9	964	9.9	206	0.38	20.6	964	0.26	7.7	206
200	35.8	200	7.7	1064	6.9	306	0.32	22.8	1064	0.18	6.6	306
400	31.5	400	5.1	1264	5.4	506	0.19	13.4	1264	0.17	6.8	506
800	25.6	800	3.7	1664	4.4	906	0.23	11.9	1664	0.15	6.2	906

^$^The cost includes acquisition of the reference designs, which is 864 EM simulations of the antenna when using feature-based optimization^50^ as listed in the table.

^#^The cost includes generation of random observables, here, 106 simulations in total to yield *N*_*r*_ = 50 accepted samples.

**Table 5:** Dual-band antenna of Fig. 4b: modeling results and benchmarking.

Number of training samples	Modeling technique											
	Kriging^32^		Nested kriging^48^		Reference-design-free modeling^49^		Feature-based nested kriging^51^			This work		
	Modeling error (%)	Model setup cost	Modeling error (%)	Model setup cost^$^	Modeling error (%)	Model setup cost^$^	Modeling error *f* (%)	Modeling error *l* (%)	Model setup cost^$^	Modeling error *f* (%)	Modeling error *l* (%)	Model setup cost
20	24.5	20	19.0	950	8.8	250	1.43	19.8	950	0.25	11.6	250
50	21.7	50	9.9	980	7.3	280	0.51	10.8	980	0.13	7.8	280
100	17.3	100	6.4	1030	5.1	330	0.39	8.4	1030	0.09	7.6	330
200	12.6	200	4.4	1130	3.8	430	0.56	6.7	1130	0.08	5.5	430
400	9.3	400	3.8	1330	3.1	630	0.43	6.3	1330	0.07	4.5	630
800	7.2	800	3.4	1730	2.5	1030	0.46	4.7	1730	0.05	3.2	1030

^$^The cost includes acquisition of the reference designs, which is 930 EM simulations of the antenna when using feature-based optimization^50^ as listed in the table. Conventional (minimax) optimization required 1201 simulations.

^#^The cost includes generation of random observables, here, 230 simulations in total to yield *N*_*r*_ = 50 accepted samples.

**Table 6:** Quasi-Yagi antenna of Fig. 4c: modeling results and benchmarking.

Number of training samples	Modeling technique											
	Kriging^32^		Nested kriging^48^		Reference-design-free modeling^49^		Feature-based nested kriging^51^			This work		
	Modeling error (%)	Model setup cost	Modeling error (%)	Model setup cost^$^	Modeling error (%)	Model setup cost^$^	Modeling error *f* (%)	Modeling error *l* (%)	Model setup cost^$^	Modeling error *f* (%)	Modeling error *l* (%)	Model setup cost
20	69.3	20	39.4	1919	17.0	212	3.3	6.33	1919	1.28	5.2	212
50	61.4	50	17.9	1949	10.8	242	2.8	6.93	1949	0.89	3.6	242
100	50.7	100	13.3	1999	8.4	292	2.8	6.55	1999	0.74	3.8	292
200	39.8	200	7.5	2099	7.1	392	2.3	6.10	2099	0.40	2.4	392
400	32.8	400	5.4	2299	5.9	592	2.4	6.53	2299	0.40	2.2	592
800	31.8	800	4.5	2699	5.0	992	2.3	5.96	2699	0.34	1.8	992

^$^The cost includes acquisition of the reference designs, which is 1899 EM simulations of the antenna when using feature-based optimization^50^ as listed in the table.

^#^The cost includes generation of random observables, here, 192 simulations in total to yield *N*_*r*_ = 50 accepted samples.

For each antenna structure, a relevant set of characteristic points has been selected: (i) the operating frequency (ring-slot antenna of Fig. 4a), (ii) two operating frequencies (dual-band antenna of Fig. 4b), and (iii) the lower and upper frequencies for which the reflection response assumes − 10 dB levels, as well as the corresponding values of the realized gain characteristic, supplemented by five additional points equally distributed in frequency in between these points (quasi-Yagi antenna of Fig. 4c). The supplementary points are required to adequately reconstruct the gain characteristics within the operating band in order to assess the antenna average in-band gain.

The results of Tables 4, 5 and 6 demonstrate that carrying out the modeling process at the level of the response features allows for achieving superior accuracy of representing the characteristic points relevant to the assumed design objectives at a remarkably small computational cost. For all the benchmark antennas, the proposed surrogate allows for achieving accuracy of less than one percent for training data sizes containing merely 50 samples from the constrained domain. Observe that the total cost of training data acquisition includes also the cost of generating 106, 230, and 192 observables for each antenna, respectively, which are necessary to define the surrogate domain. Even when taking into account these additional computational expenses, our approach requires as low as 126, 250 and 212 data samples to assess the operating frequencies of each antenna structure with the accuracy of around one percent, which makes it a very cost-efficient modeling technique. The conventional surrogate does not ensure satisfactory accuracy even for the training data set of 800 samples for two out of three benchmark antenna structures (Antenna I and III).

It should be noted that the accuracy of representing the frequency coordinates of the feature points is exceptionally good even for small data sets, whereas it is not as good for the level coordinates. On the one hand, this is of little practical significance because, for antenna design procedures, it is the frequency allocation that is of primary importance; reflection level, as long as it is below − 10 dB is of secondary relevance. On the other hand, the reason for degraded level rendition are low values of reflection coefficients at the antenna resonances (typically, − 20 dB or less), which implies a considerable amount of numerical noise caused by the EM simulation process itself (related to adaptive meshing techniques as well as terminating the time-domain simulation at relatively high levels of residual energy). The latter is corroborated by considerably better accuracy of representing the levels of the feature-points for Antenna III, which are primarily associated with the antenna gain, less affected by numerical noise due to being integral quantity.

Another observation is that both reference-design-free constrained modeling approaches (Algorithm 3 and 5) provide similar (Antenna I) or better (Antennas II and III) accuracies than both nested kriging frameworks using the set of reference designs (Algorithm 2 and 4). At the same time, the expenditures required by the proposed approach to determine the surrogate domain are significantly (from five to ten times) lower than that for the nested kriging technique.

**Figure 5:**
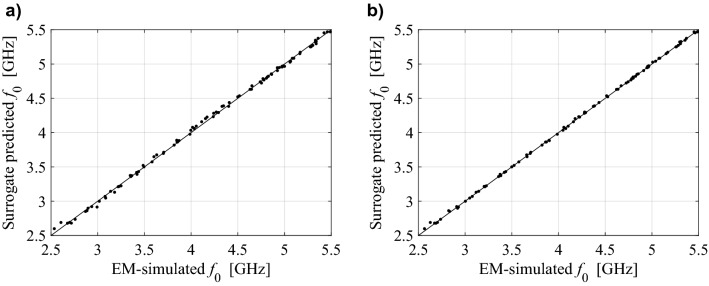
Ring-slot antenna of Fig. 4a: scatter plots of the center frequency *f*_0_ [GHz] yielded by the proposed surrogate against their EM-simulated counterparts; surrogates constructed using (**a**) *N*_*B*_ = 20 and (**b**) *N*_*B*_ = 100 samples.

**Figure 6:**
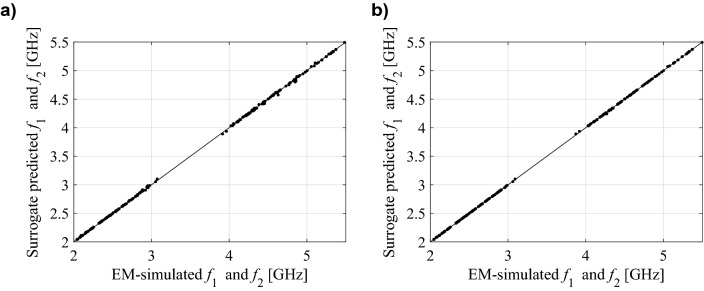
Dual-band antenna of Fig. 4b: scatter plots of the center frequencies *f*_1_ and *f*_2_ [GHz] yielded by the proposed surrogate against their EM-simulated counterparts; surrogates constructed using (**a**) *N*_*B*_ = 20 and (**b**) *N*_*B*_ = 100 samples.

**Figure 7:**
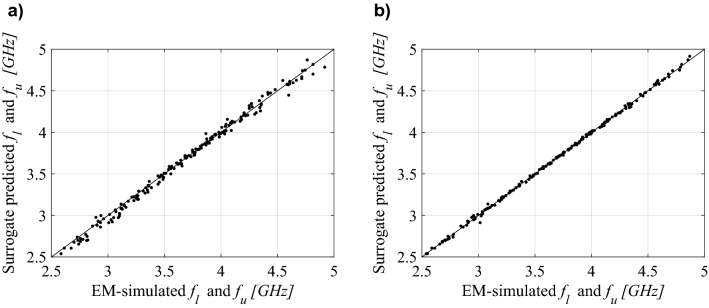
Quasi-Yagi antenna of Fig. 4c: scatter plots of the lower and upper frequencies corresponding to − 10 dB reflection levels [GHz] yielded by the proposed surrogate against their EM-simulated counterparts; surrogates constructed using (**a**) *N*_*B*_ = 20 and (**b**) *N*_*B*_ = 100 samples.

**Figure 8:**
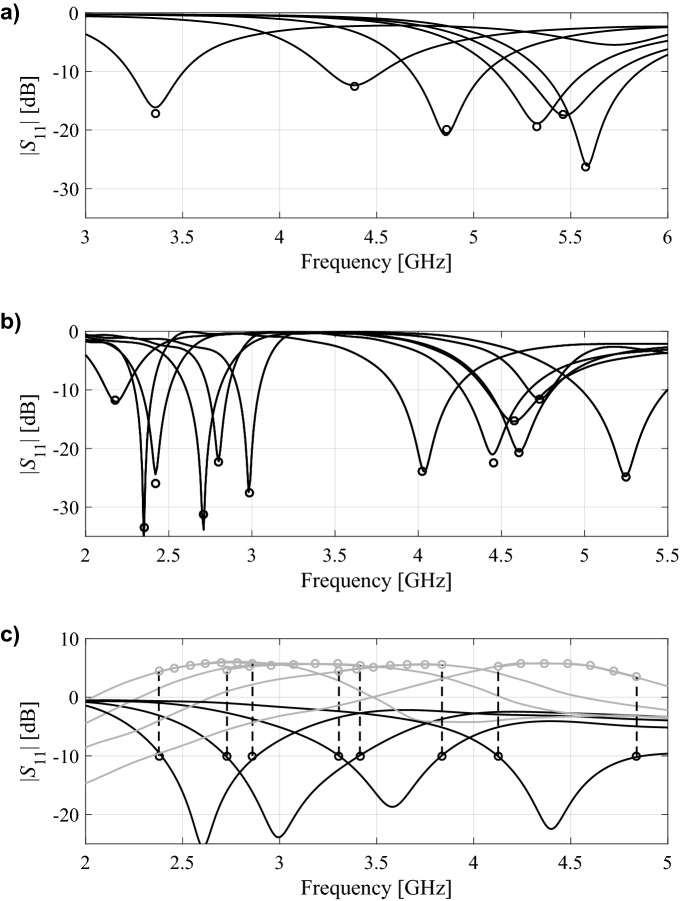
EM-simulated antenna responses at the selected test designs (—), along with the surrogate-predicted relevant antenna feature points (o); (**a**) ring-slot antenna of Fig. 4a (the operating frequency marked with circles), (**b**) dual-band antenna of Fig. 4b (the operating frequencies marked with circles), and (**c**) quasi-Yagi antenna of Fig. 4c (lower and upper frequencies corresponding to − 10 dB reflection levels, as well as corresponding values of the realized gain characteristic with five infill points are marked with circles). The surrogates set up using *N* = 200 training samples.

For better visualization, Figs. 5, 6 and 7 shows the scatter plots of the relevant feature points for all the benchmark antennas (the operating frequencies in the case of Antennas I and II, and the frequencies of − 10 dB reflection levels for Antenna III). In all cases, correlation between the surrogate-model-predicted and EM-simulated results is good, even in the case of the model set up using only 20 training data samples. For one hundred data samples, the said correlation is excellent. In addition, Fig. 8 provides also EM-simulated antenna reflection characteristics at the selected test locations, along with the characteristic points yielded by the proposed surrogate. For all antennas, the accuracy of predicting the frequencies that are relevant from the point of view of the assumed design objectives is very good.

In this work, we provided numerical verification of the predictive power of the proposed model. In engineering practice, the purpose of constructing surrogate models for antenna structures is to facilitate design procedures. In particular, the models rendered using the technique presented in this paper can be employed to optimize the antenna structures with respect to performance figures assumed as a part of the objective space. Examples include allocating the operating frequency/bandwidth at their target values, allocating resonant frequencies and improving impedance matching therein, maximizing in-band gain, as well as optimizing dimensions to achieve specific values of the operating frequencies for antenna implemented on the substrate of a specific dielectric permittivity. Application case studies have been provided in our prior works on performance-driven modelling^49,52^.

## Conclusion

This work introduced a novel approach to low-cost feature-based surrogate modeling of antenna input characteristics. The proposed surrogate is constructed in the constrained domain, which is determined cost-efficiently using a set of random observables. Our technique enhances the original reference-design-free constrained modeling framework by incorporating the response features technology, thereby allowing for further reduction of the training data acquisition cost. At the same time, it improves the surrogate model predictive power. The proposed modeling procedure has been comprehensively verified using three antenna structures. In all cases, the rendered surrogates are valid for broad ranges of geometry, material and operating parameters. Our approach has been favourably compared to several benchmark techniques: conventional data-driven model, and performance-driven methods operating on the complete antenna responses. The obtained results demonstrate that combining two algorithmic approaches, the reference-design-free model domain definition, and reformulating the modeling task in terms of characteristic points of antenna responses, enables notable computational savings without compromising surrogate model accuracy. The proposed framework may be a viable alternative to conventional data-driven procedures, especially for modeling scenarios that involve multi-parameter spaces and highly nonlinear system outputs. It is particularly suitable for constructing design-ready replacement models valid over broad ranges of operating conditions. Owing to its generic formulation, it can also find applications in various engineering fields that rely on costly simulation models.
